# Construction and genetic characterization of an interspecific raspberry hybrids panel aiming resistance to late leaf rust and adaptation to tropical regions

**DOI:** 10.1038/s41598-023-41728-8

**Published:** 2023-09-14

**Authors:** Gabriela Romêro Campos, Melina Prado, Karina Lima Reis Borges, Rafael Massahiro Yassue, Felipe Sabadin, Allison Vieira da Silva, Caio Morais de Alcântara Barbosa, Marcel Bellato Sposito, Lilian Amorim, Roberto Fritsche-Neto

**Affiliations:** 1 “Luiz de Queiroz” College of Agriculture, University of São Paulo, São Paulo, Brazil; 2 Rice Research Station, Louisiana State University AgCenter, Baton Rouge, USA; 3 School of Plant and Environmental Sciences, Virginia Tech, Blacksburg, USA

**Keywords:** Plant breeding, Plant hybridization, Next-generation sequencing, Population genetics

## Abstract

Raspberries (*Rubus* spp) are temperate climate fruits with profitable high returns and have the potential for diversification of fruit growing in mid to low-latitude regions. However, there are still no cultivars adapted to climatic conditions and high pressure of diseases that occurs in tropical areas. In this context, our objective was to evaluate the genetic diversity from a 116 raspberry genotypes panel obtained from interspecific crosses in a *testcross* scheme with four cultivars already introduced in Brazil. The panel was genotyped via genotyping-by-sequencing. 28,373 and 27,281 SNPs were obtained, using the species *R. occidentalis* and *R. idaeus* genomes as references, respectively. A third marker dataset was constructed consisting of 41,292 non-coincident markers. Overall, there were no differences in the results when using the different marker sets for the subsequent analyses. The mean heterozygosity was 0.54. The average effective population size was 174, indicating great genetic variability. The other analyses revealed that the half-sibling families were structured in three groups. It is concluded that the studied panel has great potential for breeding and further genetic studies. Moreover, only one of the three marker matrices is sufficient for diversity studies.

## Introduction

Commercial raspberry cultivars come from three main species, *Rubus idaeus* L., *Rubus strigosus* Michx., and *Rubus occidentalis* L.^1^. Despite the recent history of domestication^2^, raspberry has been gaining market share each year. For example, between 2000 and 2021, there was an increase of 209% in global production, going from 422 thousand tons to 886 thousand tons, and an increase of 132% in cultivated area, going from 83 thousand hectares to 110 thousand hectares^3^. The cultivation of raspberry in Brazil is still incipient. With several factors limiting its production, the tropical climate condition is one of the main bottlenecks. Currently, production is restricted to the country’s coldest and highest altitude regions, representing a small portion of the Brazilian arable land. However, the crop has grown in cultivation areas, due to the high added value and consequently as a good option for family farming^4,5^.

Another limiting factor is that raspberry trees suffer pressure from several diseases in low latitude regions. For example, late leaf rust (*Thekopsora americana*) can lead to losses of 30–100% of fruit and is considered the most prevalent disease in tropical conditions^5,6^. The pathogen causes premature defoliation, increases susceptibility to winter damage, and infects the fruit, making it unfit for sale in the fresh fruit market^7^. There are reports of black raspberry (*R. occidentalis*) cultivars that are highly resistant to this pathogen. Still, these cultivars are adapted to temperate regions, making cultivation in the tropics unviable.

Some raspberry cultivars of the *R. idaeus* species are adapted to higher temperatures. However, they are highly susceptible to *T. americana*^7^. Interspecific crossing between these two species has the potential to generate materials that supply the market demand since the *R. occidentalis* species is the source of resistance alleles to leaf rust and the *R. idaeus* species has greater economic importance due to favorable market characteristics. However, unilateral incompatibility and different flowering periods between these species is not synchronized^8^, hindering stable hybrid development. In this sense, studies involving interspecific hybrids provide information that allows a better understanding of raspberries and enables the exploitation and maintenance of the genetic variability of these species.

Genetic variability, interspecific and intraspecific, constitutes a source of genetic resources essential for plant breeding. It represents an allelic pool responsible for generating the existing phenotypic contrasts, resulting from the evolution of organisms in the population, arising from different processes, such as natural and artificial selection. Genetic diversity studies are essential as they seek to understand the potential resource to cope with various biotic and abiotic stresses existing or yet to come^9^. It also provides the basis for further genomic exploitation, such as Genomic Wide Association Studies (GWAS)^10,11^, and Genomic Selection (GS)^12^. Variability can be accessed in different ways, the most used currently is through SNP-type genetic markers. Genotyping by this type of marker constitutes an important tool for evaluating genetic diversity and germplasm characterization. Because it is allelic in nature and enables wide genome sampling due to the high density of markers found^13^. In this context, a panel of raspberry plants was constructed from crosses in a testcross scheme between four cultivars introduced in tropical regions of Brazil. The panel was genotyped using the *genotyping-by-sequencing* (GBS) method^14^, and, from the data obtained, we aimed to (i) estimate the genetic diversity present in the raspberry panel, (ii) understand the genetic structure present in the panel and (iii) explore the impact of the reference genome in the SNP calling process in the genetic analyses.

## Results

**Figure 1:**
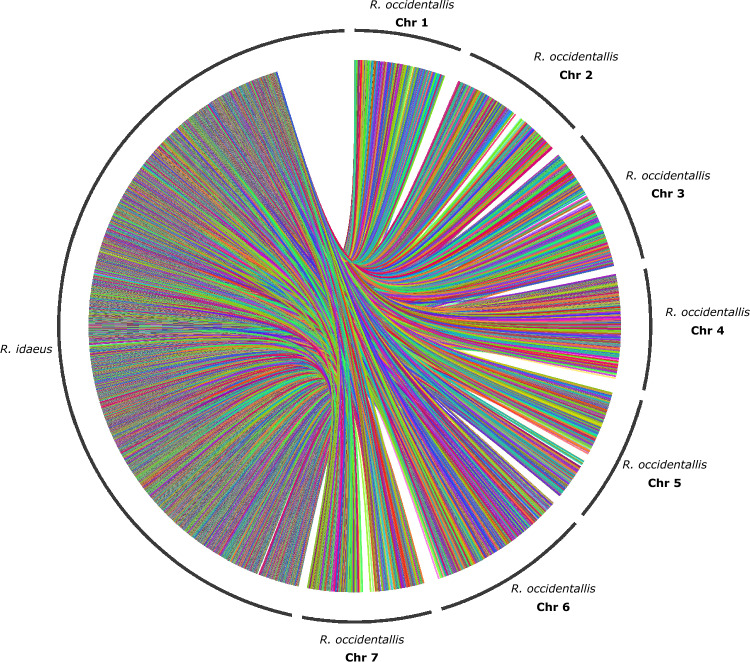
Interactions among redundant markers between *R. occidentalis* and *R. idaeus* genomes. *R. occidentalis* genome is annotated on chromosomes, and *R. idaeus* genome is annotated using scaffolds.

The diversity panel was sequenced with a mean depth of coverage of 32×. After marker filtering, 28,373 SNPs were mapped on the seven chromosomes of *R. occidentalis*, each chromosome with an average of 4053 markers, ranging from 3287 SNPs on chromosome one to 4753 on chromosome six. On the other hand, 27,281 markers were mapped to the *R. idaeus* genome and distributed across 658 scaffolds, with an average of 41 markers per scaffold. Finally, 12,919 common markers were detected in both genomes. Figure 1 shows the marker distribution across genome markers.

**Figure 2:**
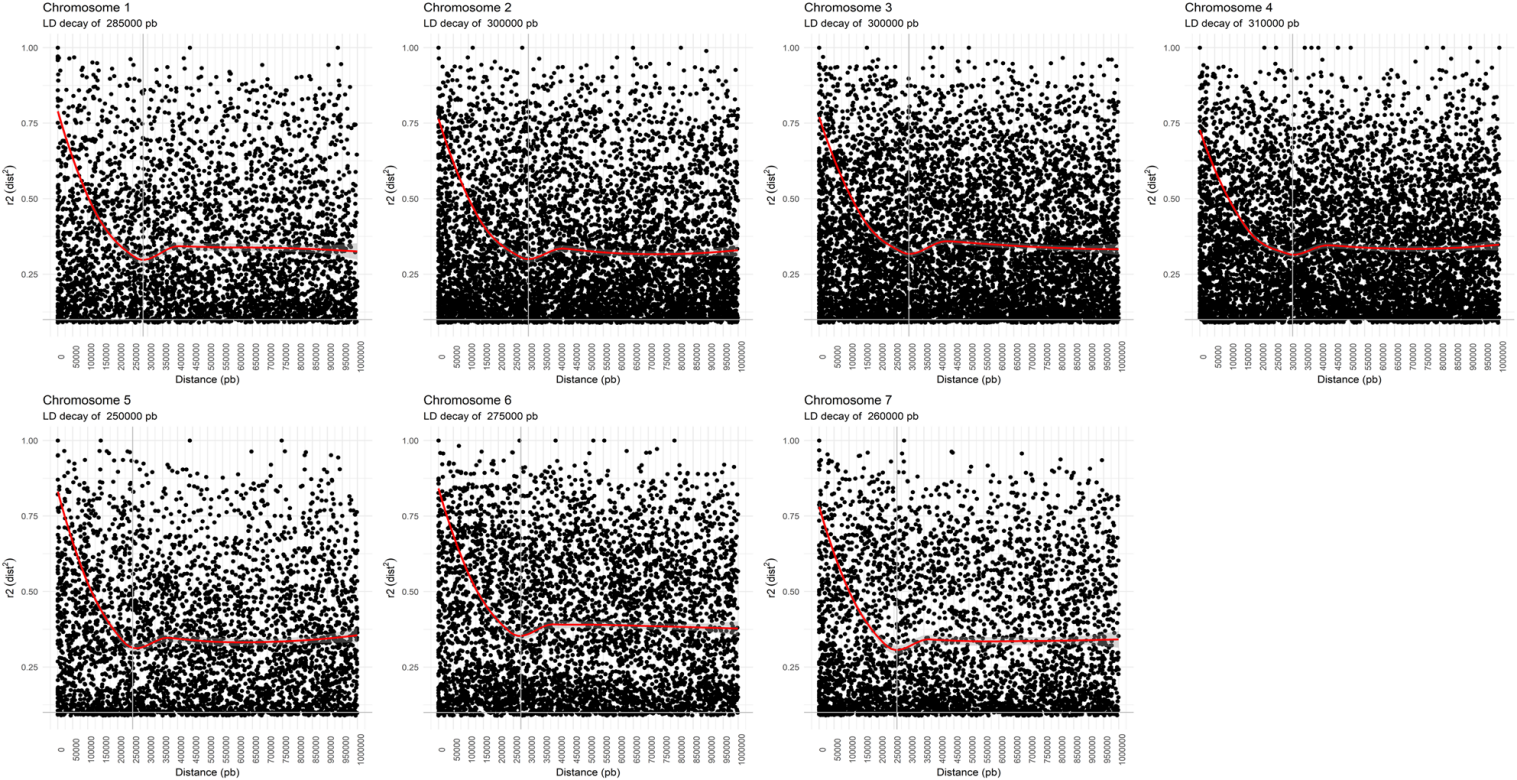
Linkage disequilibrium decay (in base pairs) of the set of markers obtained from the *R. occidentalis* genome.

Linkage disequilibrium (LD) analysis was performed using only the Moc matrix since only the *R. occidentalis* genome is structured in chromosomes^15^. The LD decay was different among chromosomes, ranging from 250 to 310 kb, considering the R2 of 0.20 (Fig. 2).

**Figure 3:**
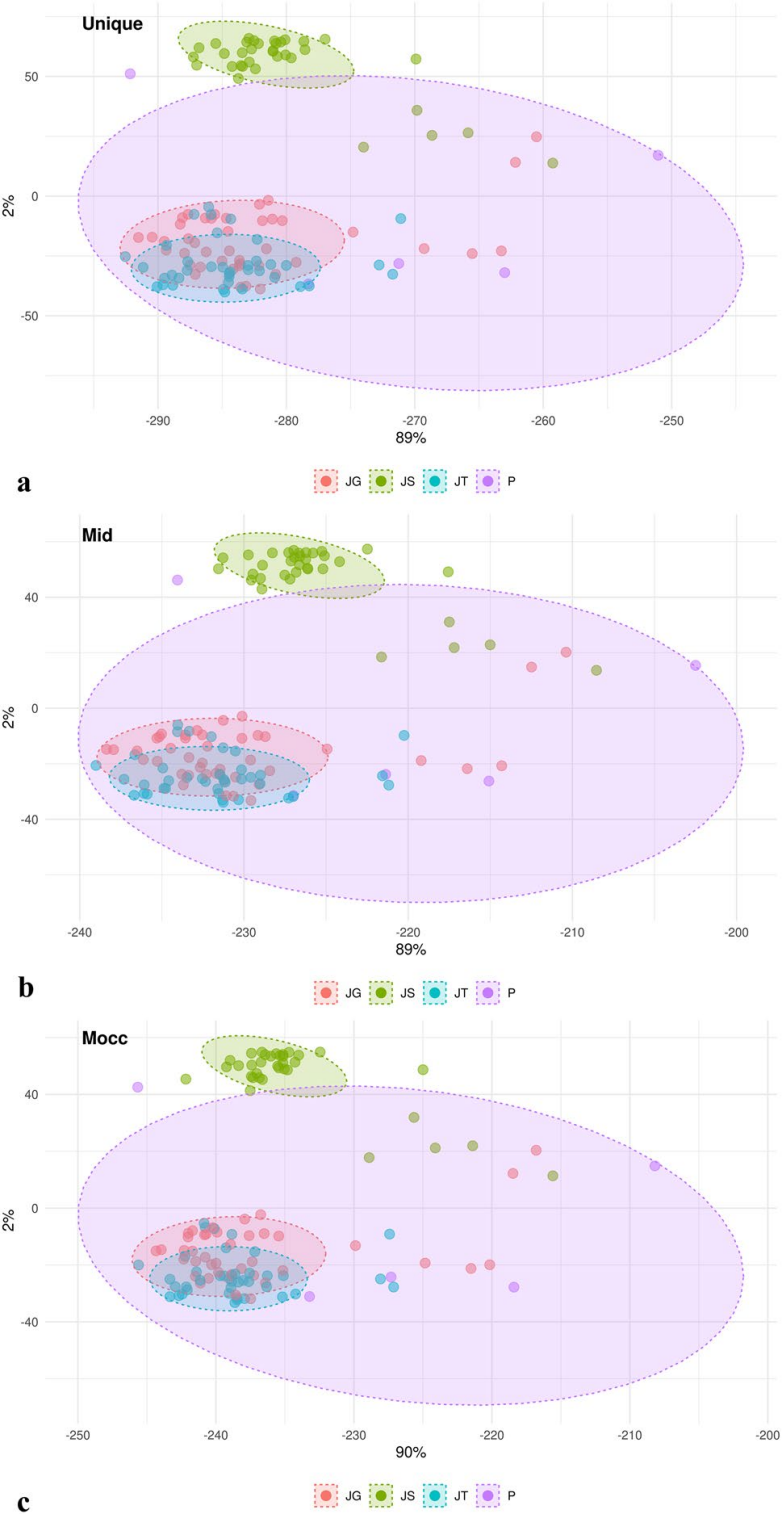
Principal component analysis considering the matrices (**a**) Unique, (**b**) Mid, and (**c**) Moc.

According to Principal Component Analysis (PCA), the first two components explained 91% of the total variation: component 1, responsible for 89%, and component 2, responsible for 2%, for the unique and Mid matrices. For the Moc matrix, the first two components explained 92% of the variation, with the first component accounting for 90% and the second for 2%. The distribution and grouping of genotypes were similar among the different matrices, distinguishing two groups, the first including most JS (Jewel × Salmon) crosses and another grouping JT (Jewel × Himbo Top) and JG crosses (Jewel × Golden Bliss) (Fig. 3).

**Figure 4:**
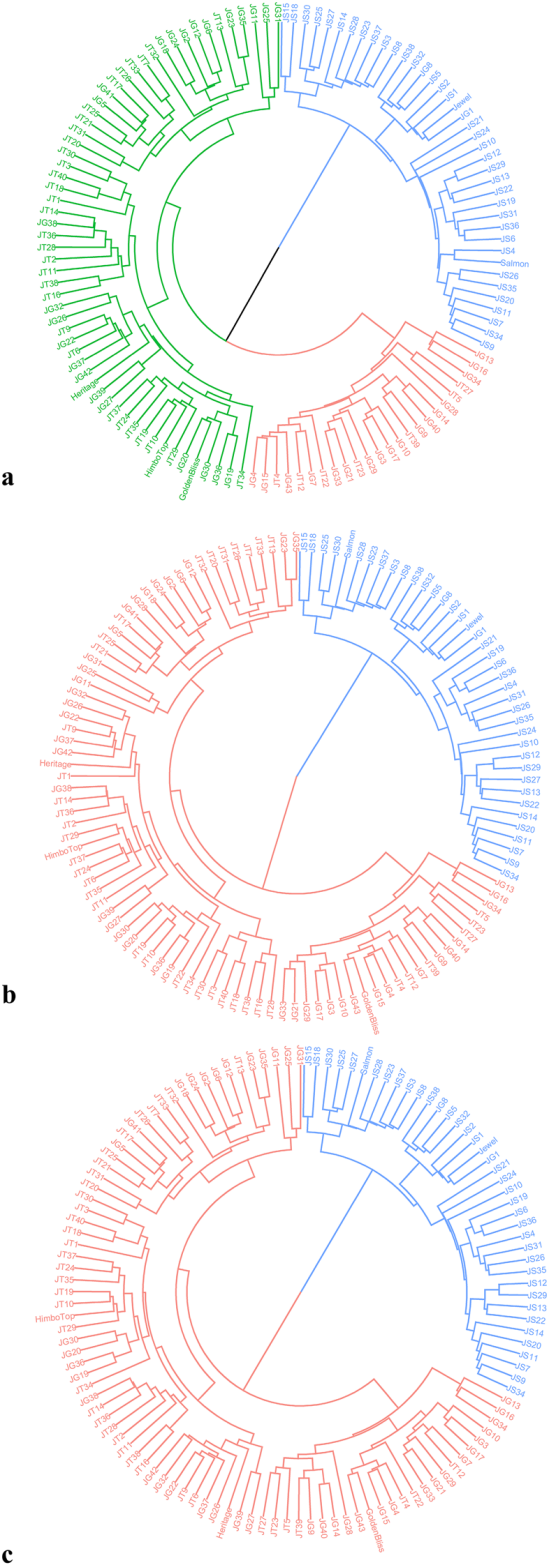
Analysis by circular dendrogram of the hierarchical clustering identified in the raspberry panel, considering the matrices (**a**) Unique, (**b**) Mid, and (**c**) Moc, where each color represents a group.

According to the dendrogram the positioning of each individual is in a likely phylogeny. Using the Mid and Moc matrices, two groups were identified, accordingly to the PCA results. However, a third group was detected using the unique matrix, corroborating the structure of half-sibling families. However, regardless of the structure, most JS progeny remained distant from the other individuals (Fig. 4).

**Figure 5:**
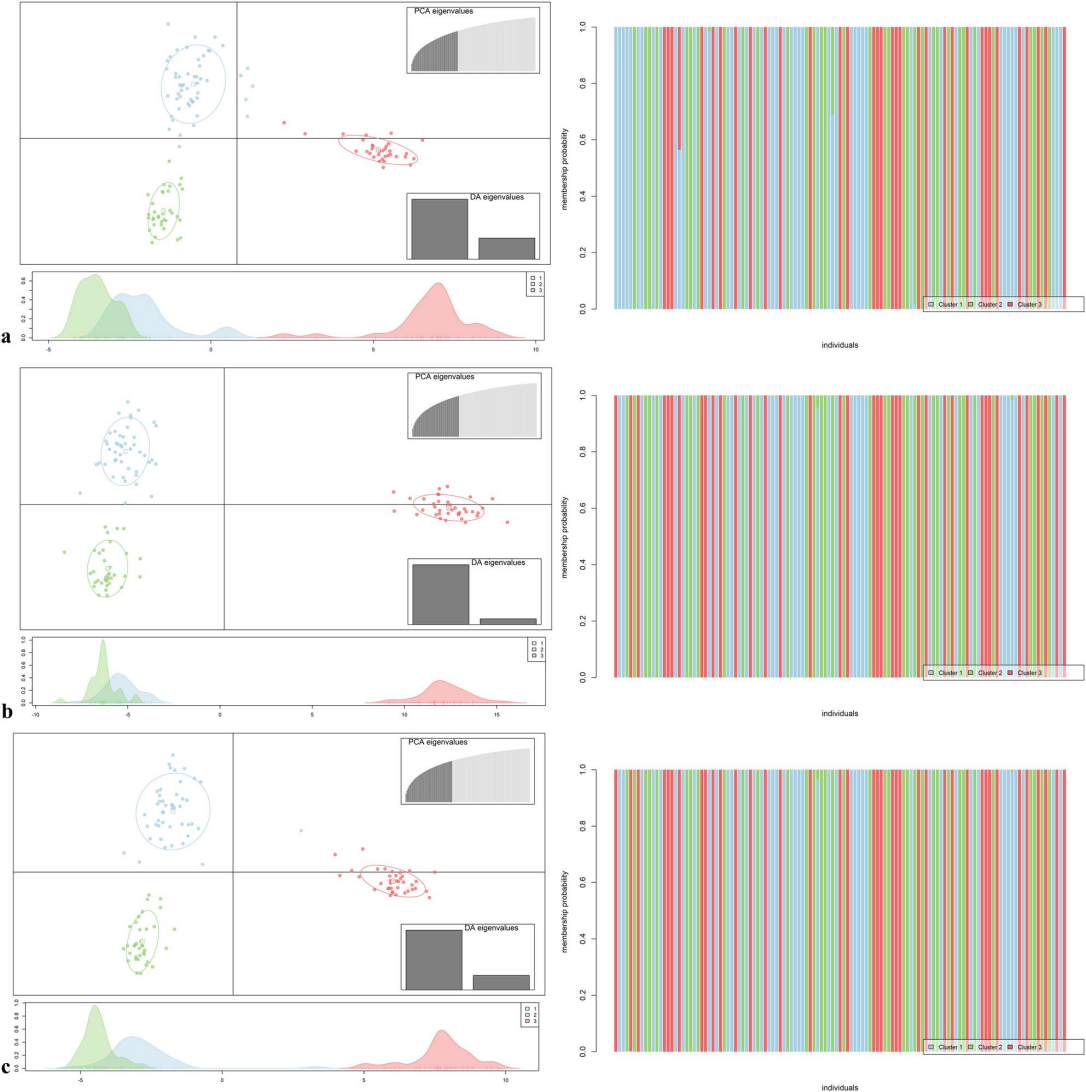
Discriminant analysis of principal components (DAPC) was performed with the 120 individuals that compose the panel, considering the matrices (**a**) Unique, (**b**) Mid, and (**c**) Moc, the right column containing the scatterplots based on two discriminant functions with three groups identified by the *k-means* method and the left column containing the graphs and the cluster probability analysis of each genotype.

DAPC analysis was performed using 45 principal components and the first two discriminant eigenvalues, which explained 74.3%, 75.7% and 76.4% of the total variance of the unique, Mid, and Moc matrices, respectively. The optimal number of groups obtained from the *k-means* analysis corresponds to three groups (Fig. 5). Regardless of the matrix, there were no significant changes in the population structure, with only some genotypes relocated from groups, and the JS progeny remained away from the other genotypes. The general population is well structured since, in most cases, each genotype was allocated exclusively to a single group (Fig. 5).

**Figure 6:**
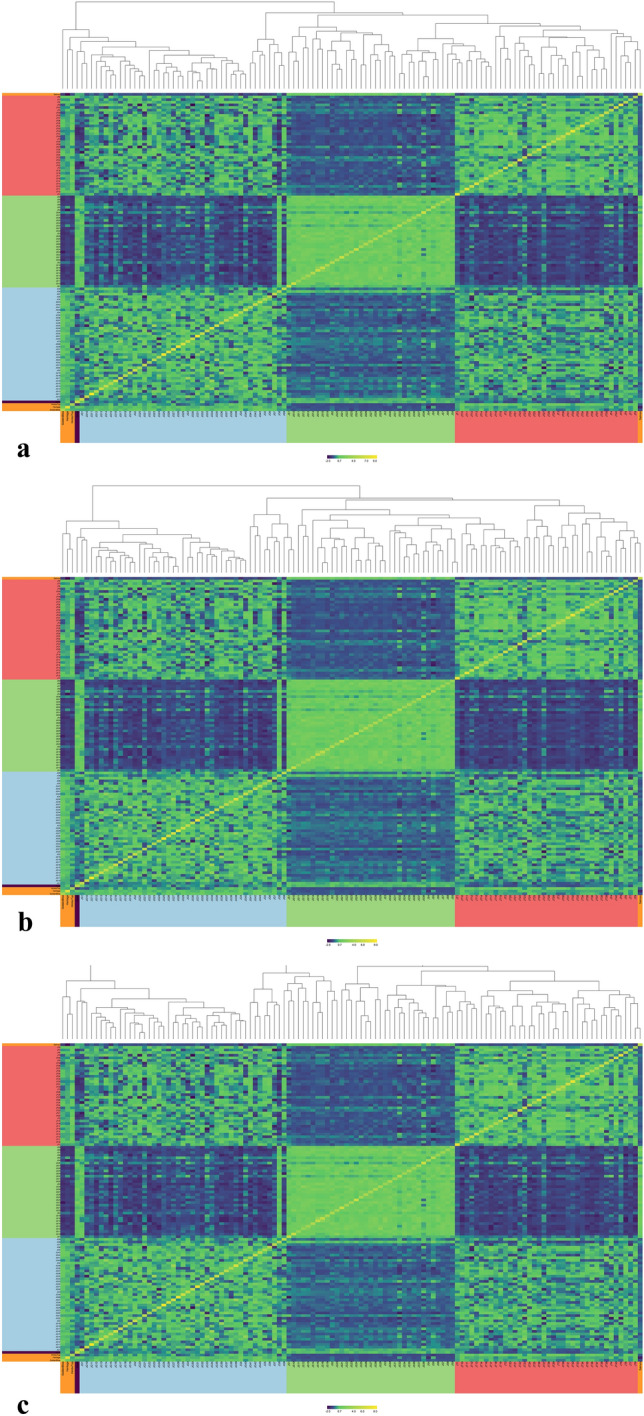
Additive relationship matrix between the genotypes that compose the panel, considering the matrices (**a**) Unique, (**b**) Mid, and (**c**) Moc, where the progenies are highlighted in blue, green, and pink, are JG, JS, and JT, respectively. Susceptible parents in orange and resistant in purple.

The heatmap of the additive genetic relationship (Fig. 6) developed using the different matrices showed the same pattern, forming three groups, one of them composed mostly of the individuals from the JS cross, as well as DAPC.

**Figure 7:**
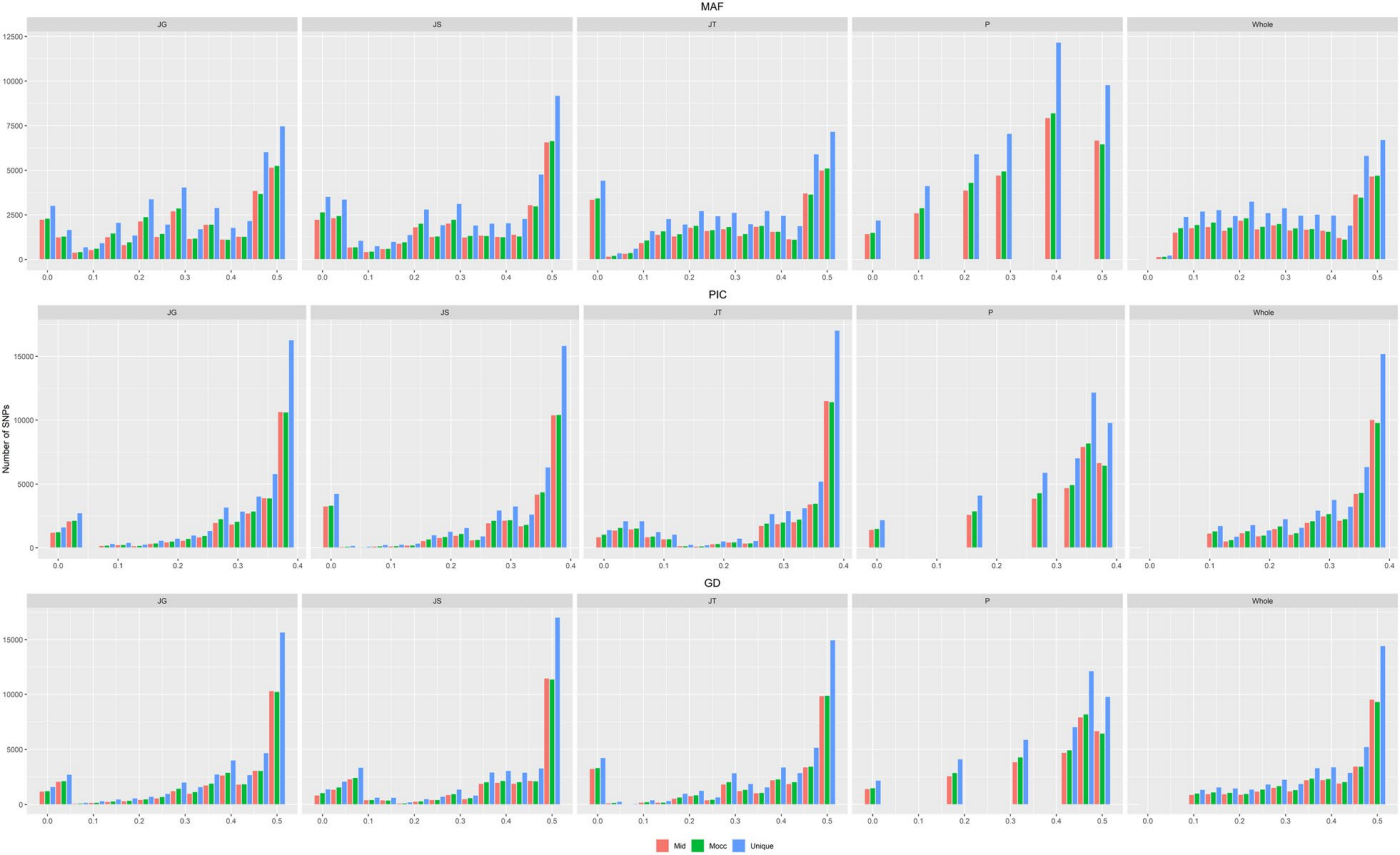
Distribution of SNPs as minor allele frequency (MAF), polymorphic information content (PIC), and genetic diversity (GD), according to the progenies JG, JS, JT, the parents, and the total SNPs.

Using the unique matrix, the values of MAF (Minor Allele Frequency) ranged from 0.05 to 0.50, with an average of 0.32. Polymorphic Information Content (PIC) values ranged from 0.10 to 0.39, with an average of 0.38, similar results were found using the Moc and Mid matrices (Fig. 7).

The estimated effective size was 174, considering the unique and Mid matrices, and 166, considering the Moc matrix. The Genetic Diversity (GD) of the individuals that make up the panel was 0.39 when using the unique and Mid matrices and 0.38 when using the Moc matrix (Table 1). The observed heterozygosity (Ho) was higher in terms of magnitude than the genetic diversity in the three situations with the unique, Mid and Moc matrices, respectively. Furthermore, the average Ho of the resistant parent was higher than the susceptible ones and their progenies (Table 1). The endogamy coefficient (*f*) was 0.35 for the unique and Mid matrix markers and 0.37 for the Moc matrix. When analyzing the progenies separately, the values remained similar, however, the resistant parent had a lower inbreeding coefficient than the other individuals in the panel. The same pattern was observed regardless of the matrix used.

**Table 1:** Mean values of diversity parameters were found using the different matrices.

Matrices	Ne	MAF	PIC	Ho	GD	F
Unique	174	0.32	0.31	0.54	0.39	0.35
Pr	2	0.39	0.36	0.77	0.48	0.21
Ps	4	0.30	0.29	0.58	0.36	0.35
JG	62	0.31	0.30	0.54	0.39	0.30
JS	51	0.31	0.30	0.54	0.39	0.35
JT	53	0.31	0.31	0.54	0.39	0.35
Mid	174	0.32	0.31	0.55	0.39	0.35
Pr	2	0.40	0.36	0.78	0.48	0.20
Ps	4	0.30	0.29	0.58	0.37	0.34
JG	64	0.31	0.29	0.55	0.37	0.30
JS	48	0.31	0.28	0.55	0.37	0.34
JT	53	0.30	0.28	0.52	0.37	0.36
Moc	166	0.31	0.30	0.54	0.38	0.37
Pr	2	0.39	0.36	0.77	0.48	0.22
Ps	4	0.29	0.28	0.57	0.36	0.36
JG	61	0.30	0.29	0.55	0.37	0.31
JS	46	0.30	0.28	0.54	0.36	0.36
JT	51	0.30	0.28	0.52	0.36	0.38

Effective population size (Ne), Minor allele frequency (MAF), polymorphic information content (PIC), observed heterozygosity (Ho), genetic diversity (GD), and inbreeding (F).

Pr: Resistant parent; Ps: Susceptible parents; JG: Progeny Jewel x Golden Bliss; JS: Progeny Jewel x Salmon; JT: Progeny Jewel x Himbo Top.

## Discussion

The development and characterization of raspberry diversity panels are crucial for the next stages of the breeding program, searching for materials more adapted to tropical climatic conditions and the late leaf rust that affects the crop^16–18^. Knowledge about the mentioned issues assists in the choice of contrasting and complementary individuals as genitors in breeding programs, thus minimizing the risks of inbreeding depression and loss of genetic variability^16,19^. In addition, it allows laying the foundation for more advanced studies on genetic control via associative mapping and genomic selection^10^.

The hierarchical grouping analysis, PCA, and DAPC showed a close relationship between the progeny JG and JT (Figs. 3, 5). One of the scenarios that may explain this proximity is a recent common ancestor between the cultivars “Himbo Top” and “Golden Bliss” which, despite artificial selection processes originating materials with distinct phenotypic characteristics, still have a great genetic similarity. A second hypothesis is that the cultivars “Golden Bliss” and “Himbo Top” come from the same cultivar but were selected separately due to mutations or segregation of important loci. The analyzes indicate that the JS progeny presents greater genetic similarity with the resistant parent compared to the JT and JG progenies (Figs. 4, 5). However, as the genealogy of the “Salmon” variety is not known, we cannot say with certainty why this is happening. However, it is possible that Salmon is a variety closer to the *R. occidentalis* species or even that the population has large epistatic selection or large inbreeding depression. Dossett et al.^16^, working with 148 cultivars of *R. occidentalis*, reported that it is challenging to distinguish cultivars due to the segregation of loci. Furthermore, breeding programs are recent^20,21^ and even the most recent cultivars have not advanced more than a few generations ahead of their ancestors^22^.

When evaluating the genetic diversity index, it is possible to identify that the panel presents a high diversity, with an average of 0.39. A study evaluating the genetic diversity of 148 genotypes of *R. occidentalis* through SSR markers, found an average GD of 0.24, a value considered high by the authors^22^. Similar values were found from the analysis of several species of the genus *Rubus*, including *R. occidentalis* and *R. idaeus*, when evaluating 10 SSR markers loci (GD = 0.23)^23^. On the other hand, higher genetic diversity (GD = 0.60)^24^ where observed when using 12 SSR markers from 48 cultivars of *R. idaeus* and *R. strigosus*.

The observed heterozygosity was higher than expected, but a similar value was observed before^16^. The high heterozygosity detected may be due to genetic recombination from interspecific crosses and the species' self-incompatibility mechanisms^25,26^. The heterozygosity observed in the panel individuals may account for the effective population size of 174, 174, and 166, considering the unique, Mid and Moc matrix, respectively, in addition to the inbreeding values. On average, 75% of the genotypes that make up the panel have heterozygosity values greater than 0.5, which can be expected due to interspecific crossings and because they are allogamous species. Concerning the parent’s heterozygosity and inbreeding, it is possible to observe that the resistant parent has greater heterozygosity than the average of the susceptible parents, and its inbreeding coefficient is lower (Table 1). A possible explanation for this result is the raspberry breeding process, which intensively selected individuals mainly based on qualitative fruit-related traits, thus, artificial selection and drift, due to the small breeding population sizes, may have decreased Ho and increased inbreeding.

In general, there was no discrepancy between the results of genomic diversity and population structure generated when the markers obtained from the alignment with the reference genome of the *R. idaeus*, the *R. occidentalis* or the set of unique SNPs from the union of the two were analyzed. Probably due to the similarity between red and black raspberries^27^.

Finally, we conclude that the panel studied has great potential for improvement and further genetic studies. In this context, aiming at the next steps in the improvement program, this study will allow the best use of available genetic resources, helping in the search for materials that are best adapted to tropical soil and climate conditions and in the identification of alleles of resistance to late leaf rust in raspberry. Moreover, only one of the three markers dataset is sufficient for diversity studies. However, differences between the marker sets are expected to be observed for association genomics or genomic prediction studies.

## Materials and methods

### Biological material

The raspberry panel consists of 116 interspecific hybrids, four parent cultivars, and a control (see Supplementary Table. S1 online). The hybrids originated from testcross scheme crossings performed in the Department of Crop Sciences, ESALQ/USP. All raspberries cultivars used for hybridization were bought in a commercial nursery at São Bento do Sapucaí, São Paulo State, Brazil (22° 41ʹ 20ʺ S; 45° 43ʹ 51ʺ W), following all applicable agricultural and genetic resource laws regarding acquisition, transport, and management of plants. The leaves of both *R. occidentalis* and *R. idaeus* were collected from cultivars, i.e. commercially cultivated varieties and obtained from a commercial nursery. The *R. occidentalis* (Black raspberry) cv. Jewel was used as a tester because it possesses late leaf rust resistance alleles^17,18^ and, as there is a unilateral incompatibility between the species^8^, it was also used as a female genitor. The selected cultivars from *R. idaeus* species (‘Golden Bliss’, ‘Himbo Top’ and ‘Salmon’) were used as pollen donors. The cultivar "Heritage" was also added to the panel. However, it was not used in the crossings, only as a check. These were chosen because they have favorable characteristics for the market, such as vigor and flavor, but are susceptible to late leaf rust^28^. Progeny size differed slightly between each cross, apparently due to issues of genetic compatibility^29^ and flowering synchronism. Finally, 43 individuals were obtained from the cross between cv. ‘Jewel’ and cv. ‘Golden Bliss’ (JG), 38 from the cross between cv. ‘Jewel’ and cv. ‘Himbo Top’ (JT), and 35 from the cross between cv. ‘Jewel’ and cv. ‘Salmon’ (JS).

### Genotyping and obtaining SNPs markers

The genotyping was performed using GBS^14^ in the Genetic Diversity and Improvement Laboratory of the Department of Genetics, ESALQ/USP. Leaf samples were collected, and the total genomic DNA was extracted using the DNeasy Plant Mini Kit (Qiagen®), following the manufacturer’s protocols. After extraction, genomic libraries were assembled following the protocol adapted from Poland et al.^30^, using two restriction enzymes: *NsiI* (New England BioLabs Inc.®) and *MseI* (New England BioLabs Inc.®).

Libraries were sequenced on a HiSeq 2500 System sequencer (Illumina, Inc), and they were aligned in Bowtie2 software^31^ with two reference genomes: *R. occidentalis* species^15^ and *R. idaeus* species^32^. SNPs were called using TASSEL 5.0 software^33^. A total of 275,904,265 reads were found, which resulted in 64,063 (*R. occidentalis*), and 64,468 (*R. idaues*) SNPs. Sequencing depth was calculated using VCFtools and quality control was performed to eliminate markers with the following parameters: non-biallelic markers, call rate > 90%, linkage disequilibrium r^2^ > 99% and MAF > 0.05. Finally, three marker matrices were constructed for the subsequent analyses, being (i) a set of unique markers originating from the alignment with the *R. occidentalis* genome and with the *R. idaeus* genome (see Supplementary Fig. S2 online), with a total of 41.292 SNPs (Unique matrix); (ii) set originating from the alignment with the *R. idaeus* genome, with a total of 27,281 SNPs (Mid matrix); and (iii) originating from the alignment with the *R. occidentalis* genome, with 28,373 SNPs (Moc matrix). For the construction of the unique matrix, it was calculated the *Pearson* correlation^34^ between the markers of the genome *R. idaeus* with the markers of the genome *R. occidentalis* and those with a correlation greater than 0.98 were considered coincident between the two genomes, being a copy removed from the set of *R. idaeus*, since the genome of *R. occidentalis* is more structured. The datasets generated and analyzed during the current study have been submitted to the Rosaceae database under the accession number tfGDR1071, which can be accessed at https://www.rosaceae.org/publication_datasets.

### Analysis of genetic diversity and structure

The marker matrices were used to study the genetic relationship between the different individuals that make up the panel. To this end, the lowest frequency allele (MAF), polymorphic information content (PIC), observed heterozygosity (Ho), and genetic diversity (GD) were calculated using the *snpReady* package^35^. In addition, using the method of Villanueva et al.^36^, the endogamy coefficient was determined (${F}_{ej}$) using Eq. (1):

1 $$Fej=\frac{{\sum }_{k=1}^{s}\frac{\left({\sum }_{i=1}^{2}{\sum }_{j=1}^{2}{I}_{ijk}\right)}{2}}{S}-1$$

where ${I}_{i{j}_{k}}$ is the identity of the two alleles ($i$ e $j$) of the individual at the SNP $k$ with value 0 for homozygotes and 1 for heterozygotes, $S$ the total number of SNPs and $N$ the total number of individuals. And from the $F,$ the effective population size ($Ne$), using Eq. (2):

2 $$Ne=\frac{1}{2Fej}$$

The additive genetic relationship matrix was obtained from the VanRaden^37^ method, and based on it, the principal component analysis (PCA) was performed. Population structure analysis was performed using the hierarchical clustering methods by Ward’s method and discriminant principal component analysis (DAPC)^38^ using the *adegenet* package^39^. Finally, the LD (${r}^{2}$) among all SNPs within less than 1 Mbp on the same chromosome. Only the Moc matrix was used because the genome of the species *R. occidentalis* is annotated on chromosome while that of *R. idaeus* is on scaffolds. A graphical representation of the markers in redundancy between the two genomes was also developed using the OmicCircos package^40^.

### Statement of methods

The procedures and techniques employed in this study involving plants were rigorously executed in strict accordance with the pertinent guidelines as outlined in the Method section. These guidelines ensured the ethical and scientific integrity of the research, promoting accurate data collection and analysis while prioritizing the proper treatment of the plant specimens involved.

### Supplementary Information

Supplementary Information.

## Data Availability

The datasets generated and analyzed during the current study have been submitted to the Rosaceae database under the accession number tfGDR1071, which can be accessed at https://www.rosaceae.org/publication_datasets.

## Code availability

All the scripts are available as supplementary information.

## References

[1] Hummer K, Hall HK, Funt RC, Hall HK (2013). Raspberries. Raspberries.

[2] Graham, J. & Brennan, R. Raspberry breeding, challenges and advances. In (eds Graham, J. & Brennan, R.) 1–16 (Springer, 2018).

[3] FAOSTAT. *Crops, rendimento e produção nos principais países produtores de framboesa*; http://www.fao.org/faostat/en/#data/QC/visualize (2021).

[4] Raseira MDCB, Gonçalves EDG, Trevisa R, Antunes LECA, Raseira MDCB, Gonçalves EDG, Trevisa R, Antunes LECA (2004). Aspectos técnicos da cultura da framboeseira. Embrapa.

[5] Caminiti A, Pagot E, Rufato ADR, Antunes LEC (2016). Produção de Framboesa. Técnicas de Produção de Framboesa e Mirtilo.

[6] Oliveira, M. E., Junior, J. S. Z., Balbino, J. M. de S., Guarçoni, R. C. & Costa, H. *Framboeseira: Cultivo e Pós-Colheita na Região Serrana do Espírito Santo* (INstituto Capixaba de Pesquisa, Assistência Técnica e Extensão Rural, 2017).

[7] Dolan A, MacFarlane S, Jennings SN, Graham J, Brennan R (2018). Pathogens in Raspberry and others *Rubus* spp. Raspberry: Breending, Challenges and Advances.

[8] Lewis D, Crowe LK (1958). Unilateral interspecific incompatibility in flowering plants. Hered. 1958.

[9] Bhadauria V (2013). Identification of *Lens culinaris* defense genes responsive to the anthracnose pathogen *Colletotrichum truncatum*. BMC Genet..

[10] Khadgi A, Weber CA (2021). Genome-Wide Association Study (GWAS) for examining the genomics controlling prickle production in red raspberry (*Rubus idaeus* L.). Agronomy.

[11] Zahid G, Aka Kaçar Y, Dönmez D, Küden A, Giordani T (2022). Perspectives and recent progress of genome-wide association studies (GWAS) in fruits. Mol. Biol. Rep..

[12] Meuwissen THE, Hayes BJ, Goddard ME (2001). Prediction of total genetic value using genome-wide dense marker maps. Genetics.

[13] Willing EM, Dreyer C, van Oosterhout C (2012). Estimates of genetic differentiation measured by FST do not necessarily require large sample sizes when using many SNP markers. PLoS ONE.

[14] Elshire RJ (2011). A robust, simple genotyping-by-sequencing (GBS) approach for high diversity species. PLoS ONE.

[15] VanBuren R (2016). The genome of black raspberry (*Rubus occidentalis*). Plant J..

[16] Dossett M, Bassil NV, Lewers KS, Finn CE (2012). Genetic diversity in wild and cultivated black raspberry (*Rubus occidentalis* L.) evaluated by simple sequence repeat markers. Genet. Resour. Crop Evol..

[17] Foster TM, Bassil NV, Dossett M, Leigh Worthington M, Graham J (2019). Genetic and genomic resources for *Rubus* breeding: A roadmap for the future. Hortic. Res..

[18] Jamieson AR, Nickerson NL (1999). Inheritance of resistance to late yellow rust (*Pucciniastrum americanum*) in red raspberry. Acta Hortic..

[19] Bhandari H, Nishant Bhanu A, Srivastava K, Singh M, Hemantaranjan A (2017). Assessment of genetic diversity in crop plants—An overview. Adv. Plants Agric. Res..

[20] Funt RC, Hall HK (2013). Raspberries.

[21] Knight, V. H. Rubus breeding worldwide and the raspberry breeding programme at Horticultural Research International, East Malling (2014).

[22] Dossett M, Bassil NV, Lewers KS, Finn CE (2012). Genetic diversity in wild and cultivated black raspberry (*Rubus occidentalis* L.) evaluated by simple sequence repeat markers. Genet. Res. Crop Evol..

[23] Lebedev VG, Subbotina NM, Maluchenko OP, Krutovsky KV, Shestibratov KA (2019). Assessment of genetic diversity in differently colored raspberry cultivars using SSR markers located in flavonoid biosynthesis genes. Agronomy.

[24] Castillo NRF, Reed BM, Graham J, Fernández-Fernández F, Bassil NV (2010). Microsatellite markers for raspberry and blackberry. J. Am. Soc. Hortic. Sci..

[25] Jennings DL (1988). Raspberries and Blackberries: Their Breeding, Diseases and Growth.

[26] Pinczinger D, von Reth M, Hanke MV, Flachowsky H (2021). Self-incompatibility of raspberry cultivars assessed by SSR markers. Sci. Hortic..

[27] Weber CA (2003). Genetic diversity in black raspberry detected by RAPD markers. HortScience.

[28] Lucero X, Wright ER, Pérez BA (2008). Occurrence of Late Leaf Rust Caused by *Pucciniastrum americanum* in Red Raspberry (*Rubus idaeus*) in Buenos Aires, Córdoba, and Entre Ríos, Argentina. Plant Dis..

[29] Keep E (2011). Incompatibility in *Rubus* with special reference to *R. idaeus* L. Can. J. Genet. Cytol..

[30] Poland JA, Brown PJ, Sorrells ME, Jannink JL (2012). Development of high-density genetic maps for barley and wheat using a novel two-enzyme genotyping-by-sequencing approach. PLoS ONE.

[31] Langmead B, Salzberg SL (2012). Fast gapped-read alignment with Bowtie 2. Nat. Methods.

[32] Wight, H. *et al.* Draft Genome Assembly and Annotation of Red Raspberry *Rubus idaeus*. *bioRxiv* 546135. Preprint at https://www.biorxiv.org/content/10.1101/546135v2. 10.1101/546135 (2019)

[33] Glaubitz JC (2014). TASSEL-GBS: A high capacity genotyping by sequencing analysis pipeline. PLoS ONE.

[34] Benesty J, Chen J, Huang Y, Cohen I (2009). Pearson correlation coefficient. Springer Top. Signal Process..

[35] Granato, I. & Fritsche-Neto, R. Package ‘snpReady’. *CRAN* (2018).

[36] Villanueva B (2021). The value of genomic relationship matrices to estimate levels of inbreeding. Genet. Sel. Evol..

[37] VanRaden PM (2008). Efficient methods to compute genomic predictions. J. Dairy Sci..

[38] Jombart T, Devillard S, Balloux F (2010). Discriminant analysis of principal components: A new method for the analysis of genetically structured populations. BMC Genet..

[39] Jombart, T. *et al.* Package ‘adegenet’. *CRAN* (2009).

[40] Hu, Y. Package ‘OmicCircos’. *CRAN* (2021).

