# Supplementary material for: Construction and genetic characterization of an interspecific raspberry hybrids panel aiming resistance to late leaf rust and adaptation to tropical regions
Source: Sci Rep. 2023 Sep 14;13:15216. doi: 10.1038/s41598-023-41728-8 (PMC10502132; doi:10.1038/s41598-023-41728-8)
Supplement: Supplementary file 1 — Supplementary Information. [file 41598_2023_41728_MOESM1_ESM.docx]

**SUPPLEMENTARY DATA**

**Construction and genetic characterization of an interspecific raspberry hybrids panel aiming resistance to late leaf rust and adaptation to tropical regions**

**Supplementary Table**

Supplementary Table 1. Size of the progenies resulting from the crossings in a *p-rep* scheme between the cultivars of the species *R. idaeus* and the cultivar of the species *R. occidentalis*

|  | Male  (*R. idaeus*) | | | Check  (*R. idaeus*) |
| --- | --- | --- | --- | --- |
| Female  (*R. occidentalis*) | "Golden Bliss" | "Himbo Top" | "Salmon" | “Heritage” |
| "Jewel" | 43 | 38 | 35 | - |

**Supplementary Figure**


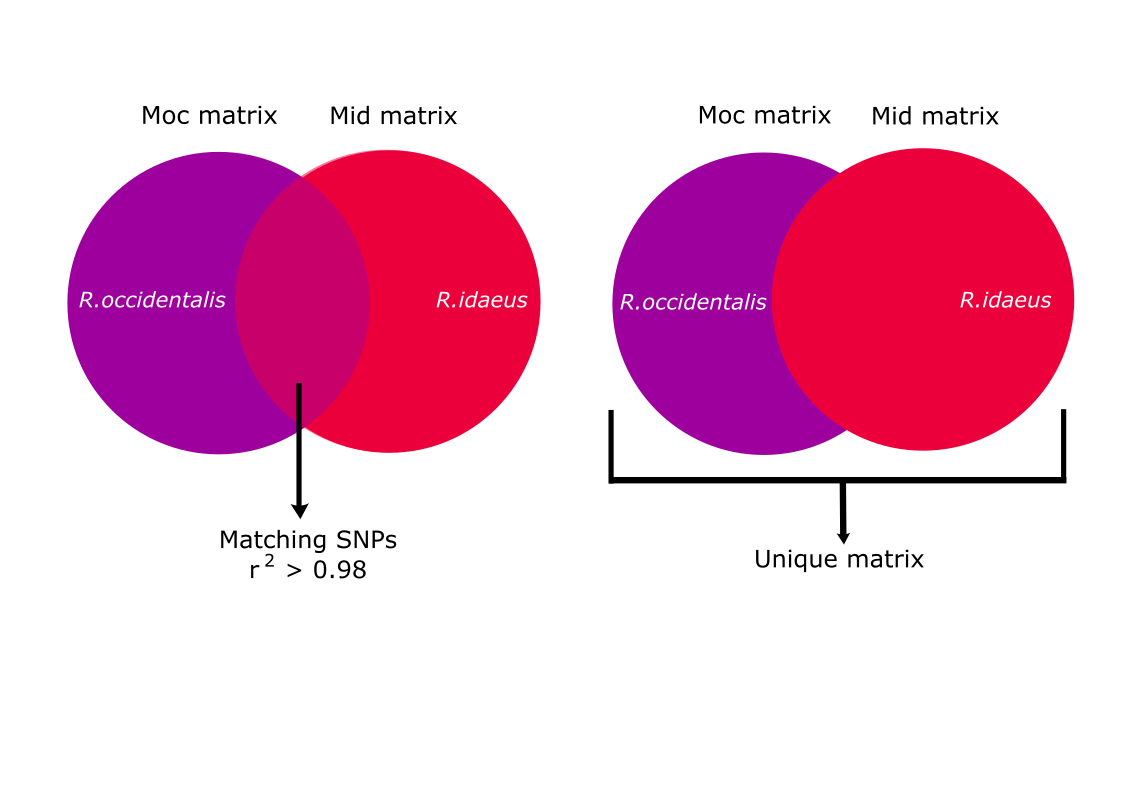
**Supplementary figure 1.** Construction of the Unique Matrix: Markers with correlation equal to or greater than 0.98 were considered coincident markers between the two genomes and were removed from Mid.


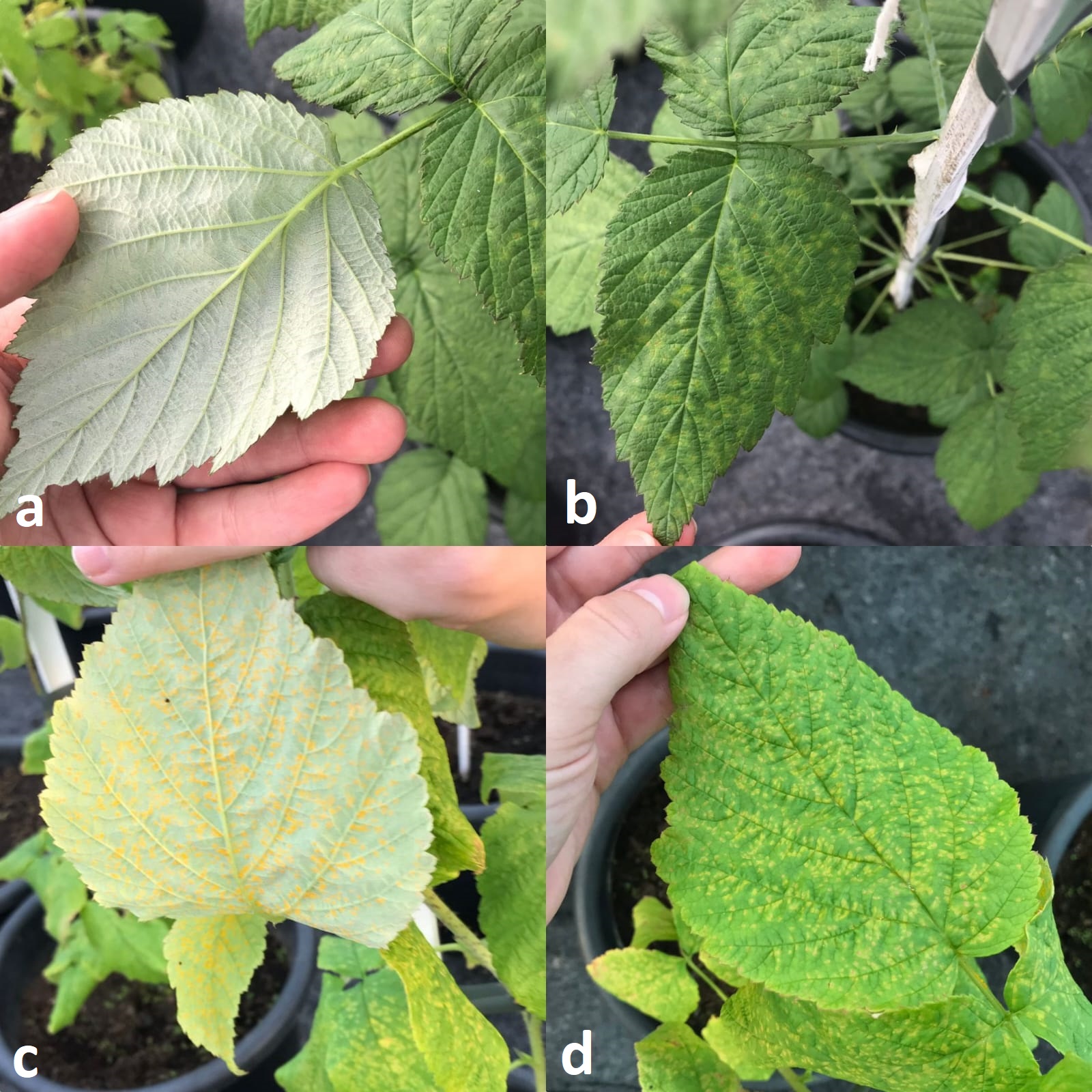


**Supplementary Figure 2.** Two parents from the diversity panel. a) Abaxial face of the cultivar “Jewel” (resistant) inoculated with rust (*Thekopsora americana*); b) Adaxial face of the “Jewel” cultivar inoculated with rust; c) Abaxial face of the “Golden Bliss” cultivar (susceptible) inoculated with rust; d) Adaxial face of the “Golden Bliss” cultivar inoculated with rust.
